# Defining & assessing the quality, usability, and utilization of immunization data

**DOI:** 10.1186/s12889-019-6709-1

**Published:** 2019-04-04

**Authors:** Peter Bloland, Adam MacNeil

**Affiliations:** 1 Strategic Information and Workforce Development Branch, Global Immunizations Division, Center for Global Health, Centers for Disease Control and Prevention, 1600 Clifton Rd, Atlanta, GA 30329 USA; 2 Strategic Information Team, Strategic Information and Workforce Development Branch, Global Immunizations Division, Center for Global Health, Centers for Disease Control and Prevention, 1600 Clifton Rd, Atlanta, GA 30329 USA; 3 Surveillance, Epidemiology, & Monitoring and Evaluation Team, Global Tuberculosis Branch, Division of Global HIV and TB, Center for Global Health, Centers for Disease Control and Prevention, 1600 Clifton Rd, Atlanta, GA 30329 USA

**Keywords:** Data quality, Data use, Immunization program, Immunization information, Low and middle-income countries

## Abstract

**Background:**

High quality data are needed for decision-making at all levels of the public health system, from guiding public health activities at the local level, to informing national policy development, to monitoring the impact of global initiatives. Although a number of approaches have been developed to evaluate the underlying quality of routinely collected vaccination administrative data, there remains a lack of consensus around how data quality is best defined or measured.

**Discussion:**

We present a definitional framework that is intended to disentangle many of the elements that have confused discussions of vaccination data quality to date. The framework describes immunization data in terms of three key characteristics: data quality, data usability, and data utilization. The framework also offers concrete suggestions for a specific set of indicators that could be used to better understand immunization those key characteristics, including Trueness, Concurrence, Relevancy, Efficiency, Completeness, Timeliness, Integrity, Consistency, and Utilization.

**Conclusion:**

Being deliberate about the choice of indicators; being clear on their definitions, limitations, and methods of measurement; and describing how those indicators work together to give a more comprehensive and practical understanding of immunization data quality, usability, and use, should yield more informed, and therefore better, programmatic decision-making.

## Background

High quality public health data are needed for many reasons, including decision-making and planning at all levels of the health system, monitoring program performance, and justifying financial support; this is equally true for data related to vaccinations [1, 2]. The primary way that the performance of national immunization programs is monitored and evaluated on a global level is by tracking national estimates of vaccine coverage (the proportion of a target population that received a given vaccine) over time [3]. A key source of information for generating these estimates is routinely collected data on numbers of vaccines administered (called aggregate or “administrative” data) [3].

Unfortunately, such data from low- and middle income countries are often deemed to be of poor quality [3–6]. Because of the need for high quality data, many organizations have invested considerably in improving data quality, including Gavi, The Vaccine Alliance (Gavi); the World Health Organization (WHO); UNICEF; and the U.S. Centers for Disease Control and Prevention (CDC). Unfortunately, there is no gold standard measurement of data quality against which any other measurement can be compared.

Some published methods for assessing the quality of routinely collected (administrative) data at national and subnational levels include the immunization Data Quality Audit (DQA), the immunization Data Quality Self-Assessment (DQS)*,* and WHO’s Service Availability and Readiness Assessment (SARA) [7–9], however each has important limitations, as described later. In order to identify a more comprehensive range of issues that might affect data quality, CDC, WHO, and country-level partners have developed a holistic approach that looks broadly at the immunization information system itself, including triangulating data from multiple sources and looking at other relevant components (e.g., workforce) that affect the overall quality of vaccination coverage data [10].

On a global level, national vaccine coverage estimates derived from administrative data quality are frequently evaluated by comparing estimates of vaccination coverage from administrative data with coverage estimates from population-based surveys. This approach may reveal large differences in coverage estimates; a recent comparison of country-level coverage estimates derived from administrative data with those derived from representative surveys found that the administrative data were 26–30% higher than estimates from surveys for the same year [11]. While coverage estimates derived from surveys are considered the most reliable, data sources used to determine vaccination status of children (e.g., parental recall, review of home-based records) are themselves subject to inaccuracy [12, 13]. Although discordance between administrative data and survey-based estimates might indicate underlying issues with data quality for at least one of these sources, data quality issues can occur with both, so good agreement between sources does not necessarily guarantee high quality data. Furthermore the accuracy of administrative vaccine coverage relies on both accurate administrative data *and* accurate estimates of the target population, introducing an additional source of potential error [14].

To adjust for potentially poor quality data, WHO and UNICEF have developed the WHO/UNICEF Estimates of National Immunization Coverage (WUENIC) approach that yields a “best guess” estimate of national vaccination coverage by reviewing information from official country reports, surveys, temporal trends, and expert judgement [15]. The WUENIC approach provides relevant contextual information (such as known vaccine stockouts) and a subjective grade of confidence in the quality of the data sources used. An alternative approach proposed by the Institute of Health Metrics and Evaluation (IHME) uses a model-driven quantitative approach and provides statistically derived 95% uncertainty intervals [16]. Ultimately, though, assessment of administrative data quality represents a comparison of differences between potentially flawed measures with little way to judge the magnitude or importance of those flaws.

Challenges also exist in simply defining “data quality” and consensus around a single definition is lacking [1]. Certain terms (such as accuracy, reliability, precision, and validity) are used interchangeably in discussions of data quality even though they actually mean different things (see Table 1) [1].

**Table 1:** Definitions of terms often used with regard to immunization data

Term	Definition	References
Trueness (also “accuracy” and “unbiasedness”)	Closeness of a measurement or estimate to the exact or true value of the thing that was intended to be measured; (N.B.: ISO definition further specifies accuracy being combination of both “trueness” and precision)	[17, 23–25]
Concurrence (or “congruence”)	Degree of agreement between different methods intended to measure the same thing	
Precision	Degree of spread of a series of observations or measurements - combination of repeatability and reproducibility; how tightly the distribution of an estimator clusters about its center; degree of being free of random error	[17, 24, 26]
Reliability (or “consistency”)	Repeated estimates/measurements produce similar results under similar conditions; the closeness of the initial estimated value(s) to the subsequent estimated values	[17, 24]
Repeatability	Degree of agreement (variation) of a measurement under constant conditions using the same instrument with the same operator over a relatively short period of time	[17]
Reproducibility	Degree of agreement (variation) of a measurement under non-standardized conditions, i.e., same measurement method but conducted by different operators over longer periods of time.	[17, 23]
Usability	Degree to which data are of sufficient quality (accuracy), completeness, timeliness to allow for effective decision making	
Utilization (or “Use”)	Degree to which data are actually used in decision-making	
Validity	Degree to which an assessment measures what it is intended to measure; degree of being free of systematic error	[17, 24, 27]

Applying inconsistent and vague definitions has made measuring the underlying quality of administrative data subject to incorrect or misleading interpretation. For example, the prevailing methods for formally assessing vaccination data quality, the DQA and the DQS, focus heavily on evaluating the consistency of data between sources (especially between facility-based paper records and aggregated monthly reports) or between various reporting levels (such as between facility, district, and central levels) [7, 8]. However, the point of primary data collection – by the frontline healthcare worker at the time of vaccination – is mostly unexamined, yet potentially represents a substantial source of error that, once recorded, is not correctable at higher levels of the system. The DQA calls for observation of vaccination sessions, but only calls for observing five healthcare worker-patient interactions per facility if visits coincide with vaccination sessions [7]. The DQS does not address primary data collection at all, but instead defines “accuracy” as consistency of data between reporting levels [8].

## Proposed definitional framework

We believe that basic concepts underlying “data quality” need to be untangled in order to have more operationally relevant discussions about what each means; how they interact with each other; the relative importance of each; and appropriate methods with which to measure, interpret, and improve data quality.

We therefore offer a framework that describes immunization data in terms of three key characteristics (data quality, data usability, and data utilization) intended to keep the underlying concepts separate and specific; to keep the definition of each concept as understandable and measurable as possible (or to explicitly identify those that are inherently difficult to measure); and to provide specific indicators and methods that can be used for assessment.

### I.Data quality

The characteristic of data quality focuses on two indicators directly related to the actual underlying quality of the data: trueness and concurrence.

**Fig. 1:**
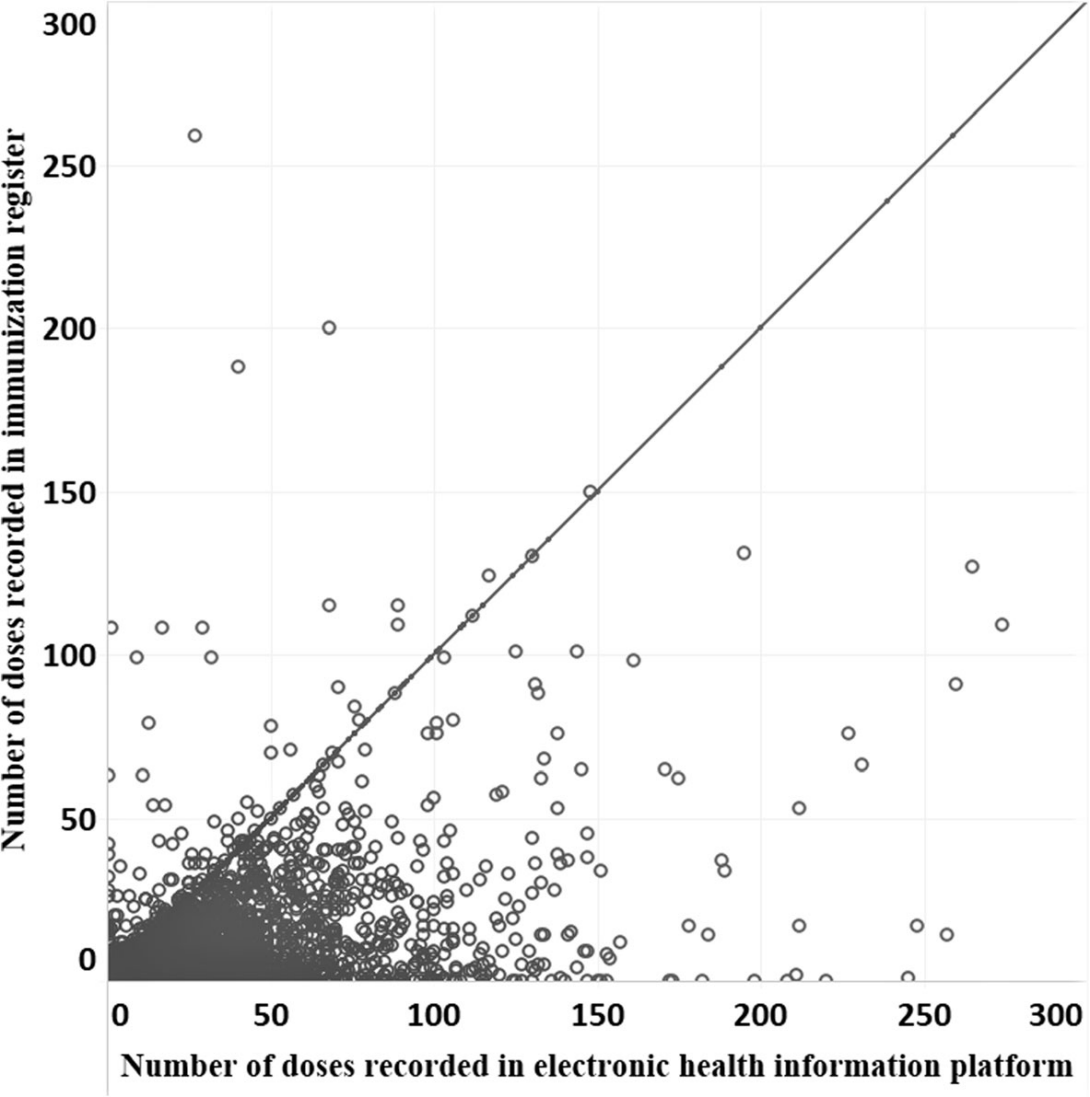
Doses of DTP3 recorded on national electronic health information platform compared to facility-based register from 1549 health facilities, Uganda, 2014–2016. Each dot reflects data from an individual facility (Ward et al., unpublished data, limited to facilities reporting less than 300 doses)

1. Trueness – The International Organization of Standardization (ISO) defines “trueness” as a measure of the degree of agreement between a given measurement and the actual (true) value [17]. For immunization data, this indicator is the most difficult to measure and often is only referred to indirectly, if at all. While comparison measures, such as comparing a paper health facility report to digital data for the health facility, are useful for evaluating discrepancies between sources, it is not possible through this approach to understand the true number of vaccinations that were given.

- *Measurement:* In the context of data quality, “truth” would reflect the actual number of vaccines given to individuals in a given period of time. “Trueness” would reflect the difference between “truth” as defined above and what is recorded by a health care worker in whatever instruments are in use (e.g., immunization register or tally sheet). The most straightforward approach to measuring trueness would be an observational study where immunization sessions are observed and the health care workers’ records are compared to those of the observer. Although straightforward, this approach could at least theoretically introduce bias due to the Hawthorne effect [18]. Other evaluation approaches are possible, many adapted from the quality of care literature, but would require various methods to minimize this issue [19]. However, given the time and effort these designs imply, it is unlikely that evaluation of trueness of data would be conducted routinely.

2. Concurrence – Vaccination data are often entered in multiple instruments, including daily tallies of vaccines given, facility-based child immunization registers, aggregated monthly reports, and home-based child health cards, resulting in multiple potential data sources that could be used to estimate the number of doses administered and, ultimately, coverage. “Concurrence” measures the degree to which administrative data obtained from different sources agree with each other. However, because none of these can be considered a gold standard, an assumption is made that strong agreement (“concurrence”) between different data sources suggests that data are close to the “truth” while a low degree of agreement indicate that the data are error-prone, and therefore of low quality. A high degree of concurrence between data sources does not, however, guarantee trueness of the data.

- *Measurement:* Concurrence is measured by comparing data from multiple sources, such as the health facility registry, tally sheet, health facility aggregate report, and digital data in the country’s information system. Figure 1 presents an example of plotting the number of children receiving their 3rd dose of diphtheria, pertussis, tetanus containing vaccine (DTP3) by health facility in Uganda from 2 different sources (e.g, facility register and national electronic health information platform); if there is complete concurrence between data sources, data points for the given health facility would fall on the 45-degree line. This indicator might also be useful in identifying data sources that have the most potentially useful data. For example, in much of Africa, child health registers are often used in parallel to tally sheets for collecting information on vaccines administered. Tally sheets often show higher numbers of vaccines given than child health registers [20]. Given the difficulty of using paper-based child health registers compared with relatively simple tally sheets, the data in registers are likely to be poor or incomplete while tally sheets may be closer to the “truth”.

### II. Data usability

The characteristic of usability adds indicators that describe the data’s suitability for decision-making and program management, specifically that: (1) all necessary data are collected (relevancy), (2) the information system is not overloaded with additional data that have limited usefulness (efficiency), (3) data are complete (completeness), (4) data are available to be used when needed (timeliness), (5) data at each level of the health system are unchanged from those originally recorded at the point of primary collection (integrity), and (6) data are free of large and unpredictable fluctuations (consistency).

Associated indicators are:

1. Relevancy – This indicator measures the degree to which the data reflect what is most important for supporting decision-making.
2. Efficiency – Efficiency is the degree to which data focus *only* on what is most important for decision-making and that the system tasked with collecting those data is not burdened with large amounts of data with limited relevance. Collection of data with limited additional use for decision-making and/or use of redundant, parallel systems for entering and reporting the same data can have substantial impact on staff time, adding unnecessary burden to the immunization program, which, in turn, can affect quality of those data.

- *Measurement:* Assessing relevancy and efficiency is a matter of reviewing the data elements being collected in a given immunization information system and assessing them for their potential usefulness in decision-making. Relevancy and efficiency imply the existence of an agreed-upon minimum set of non-redundant data points required for appropriately informed decision-making and program management, although consensus does not currently exist around what would constitute such a minimum data set.
  3. Completeness – Completeness is an indicator that reflects whether or not all relevant data needed for decision-making are available for use.

*Measurement:* There are two separate components implied by this indicator: (1) the proportion of all data points that are supposed to be entered in the official record at the point of primary data collection that are recorded and reported (“completeness of data”), and (2) the proportion of all sites that should be reporting that are doing so during a specified period of time (“completeness of reporting”). Assessing completeness of reporting requires that a current census of all sites that provide vaccination services exists (including non-governmental and private sector sites). Completeness is an indicator that has been frequently used within existing definitions of data quality, however has tended to refer only to completeness of reporting [7, 8]. We believe it is important, when using the term ‘completeness’ to clearly differentiate between these 2 component measures.

4. Timeliness – Timeliness reflects the degree to which data are current and available when needed to inform decisions.

- *Measurement:* This indicator measures the proportion of reports that were delivered to the next level of the reporting system within a specified amount of time as defined by the program. Timeliness is an indicator that is frequently used within existing definitions of data quality [7, 8], however, within this proposed framework, it is associated with data usability rather than quality.
  5. Integrity – Many information systems initially collect data on paper forms (e.g., daily tally sheets, child health registers) that are collated into an aggregate report (e.g., a facility’s monthly report) and then transcribed into an electronic data base at a higher level of the system (such as the district) and transmitted onward. At each point, possibility of mathematical error or incorrect transcription exists. Because evaluation of performance is often linked to financial resources, there can be an incentive to inflate numbers in order to meet expected targets. Integrity of data, therefore, reflects the degree to which data, once entered into the official record, are lost, incorrectly transcribed from one record to another, or otherwise altered from the original.
- *Measurement*: Methods for measuring data integrity are well described in existing methodologies, especially the DQA [7]. Data entered in the primary record should remain unchanged as those data are aggregated, transcribed, and reported to the next higher level. An assessment of data integrity would include comparisons of records to confirm that no mathematical errors were introduced as daily data were aggregated into a facility’s monthly reports, that those data were then transcribed correctly when entered into a data base at district level, and no other changes were introduced as data moved from district to regional to central level or from the country level to the global level.
  6. Consistency – In order for data to be of maximal value, the underlying characteristics of those data must remain relatively free of dramatic and unpredictable variation. If doses of vaccine reported by an administrative unit are consistently inflated, a drop in doses reported in an individual month could still be of value to initiate action by supervisors, even if the actual number of doses reported is not true. In contrast, if data completeness or integrity fluctuates dramatically month to month, it would be hard to determine if any given change represented a true change in program performance. Maintenance of consistency requires both a workforce component for collecting and reporting data in a consistent manner, as well as an operations component that ensures consistent availability of paper-based tools and support for digital systems, if used.
- *Measurement*: An assessment of trends in program performance over time should provide clues to the consistency of data. While it may not be possible to assess the consistency of data fully, indicators of inconsistent data may be represented by quantitative observations (e.g., large changes in numbers) or qualitative observations (e.g., facilities reporting stockouts of paper tools).

### III. Data utilization

This characteristic adds evidence that data are actually being *used*. Measurement of data utilization requires either prospective tracking of how data are used to make decisions or retrospective investigation of the degree to which data were used in making a given decision.

## Discussion

Although we have focused on issues related to vaccines, the underlying issues likely hold across many public health programs. At the center of the push for high-quality data is a search for trueness in available data and the estimates derived from them. For vaccine data, in settings where a high degree of attention is paid and value ascribed to careful record keeping, where there is a good system of accountability, and where there are accurate estimates of the target population, routinely collected immunization data likely can be trusted. However, in many low- and middle-income countries, this is often not the case.

**Fig. 2:**
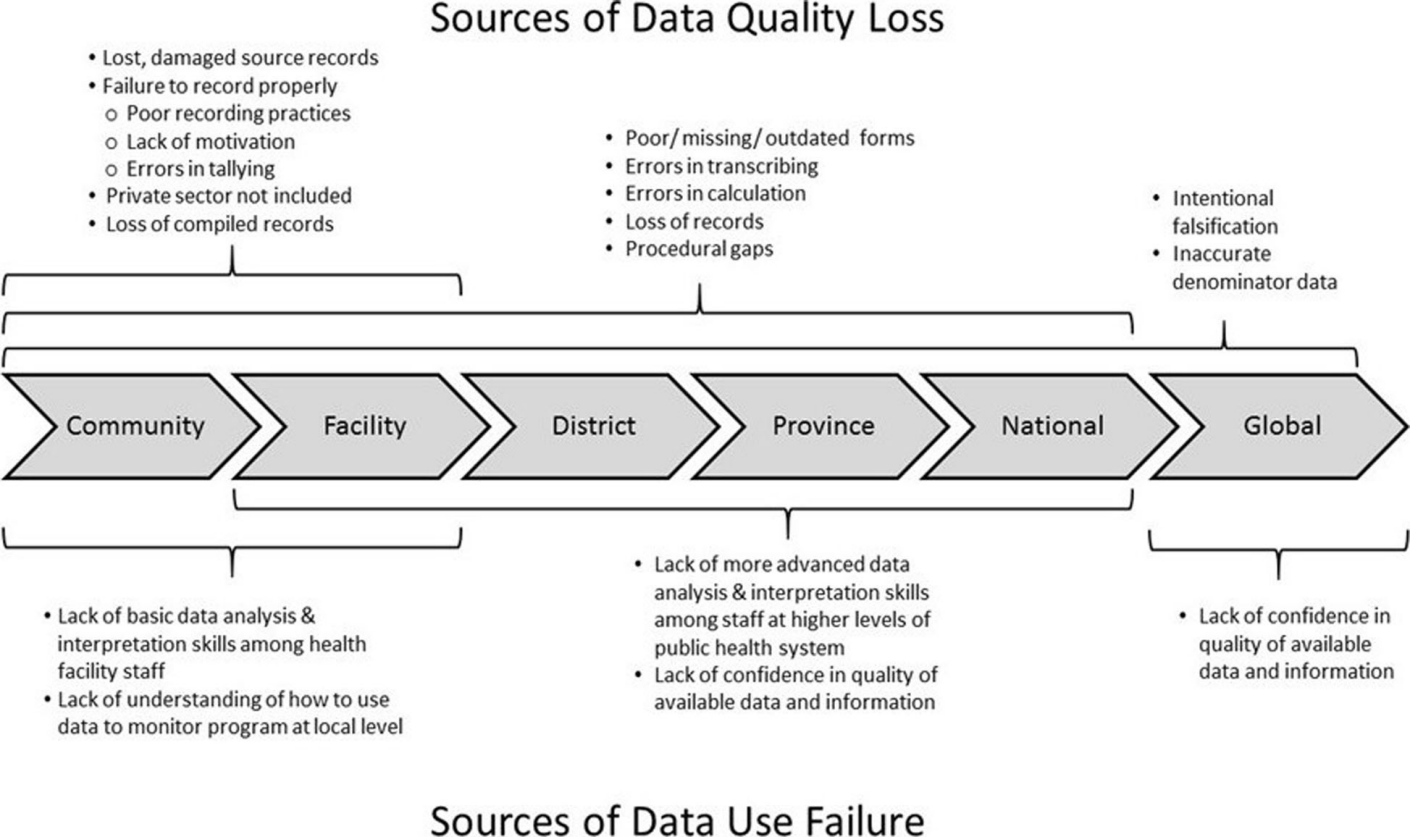
Possible sources of data quality loss and data use failure as administrative data progress from primary point of collection to the level of global reporting

To improve data quality overall, concern about data must begin at the point where they are first collected (Fig. 2). Those responsible for primary data collection (typically frontline health care workers) are often overworked, under-motivated, or see data recording duties as unwanted, time-consuming additions to providing care to patients [21]. Forms needed to collect data properly are frequently outdated, poorly designed, or missing. Because there is often 1) little or no feedback on the data they do report, 2) limited time and ability to analyze data locally, or 3) a lack of understanding of how to use the data to help them do their jobs, they often do not see the value of the data they are charged with collecting [21]. Finally, there are rarely consequences for poor data recording practices or rewards for good record keeping; in some cases, there may actually be incentives to falsify reported data [3, 8, 16]. If there is not adequate attention paid to the quality and consistency of initial data collection, no technologic solution or amount of effort invested after the fact will improve those data.

Nonetheless, good decisions can be made with bad, or even no, data, while bad decisions can be made despite good data being available. Therefore, what degree of quality (especially if narrowly defined as “trueness”) is actually required to make informed or “good” decisions, to plan, or to monitor program performance? Should the international community be focusing on improving data quality in order to meet its own information needs while mostly ignoring whether or not data are actually being used at country and local levels to monitor and improve service delivery?

We believe that while data do need to have a reasonable level of quality, a focus on quality alone (or even quality and usability, as defined here) is shortsighted and misses the value and importance of data utilization. Any real, lasting improvements in data quality will likely not be achieved through external pressure from the international community, but rather through increasing the internal demand for data, especially at the level of primary data collection. Improving the quality and usability of routinely collected immunization data will likely depend on a feedback loop being established where increasing use of data (especially at or near the point of primary collection) results in an increased demand for better data that, in turn, encourages more use [22]. Such a feedback loop would require far more attention be paid to increasing data use skills of staff throughout the health system, establishing standard operating procedures for primary data collection, simplifying reporting, introducing accountability structures and procedures, and implementing automated data support and visualization systems where possible. With such an effort, we believe that not only would the quality and usability of immunization-related data be greatly improved, but more importantly, so too would the utilization of those data, leading to better decision-making for immunization program management and improved health outcomes.

## Conclusion

Being deliberate about the choice of indicators; being clear on their definitions, limitations, and methods of measurement; and describing how those indicators work together to give a more comprehensive and practical understanding of immunization data quality, usability, and use, should yield more informed, and therefore better, programmatic decision-making.

## Abbreviations

CDC: United States Centers for Disease Control and Prevention
DQA: Data Quality Audit
DQS: Data Quality Self-Assessment
DTP3: 3rd dose of vaccine for diphtheria, pertussis, tetanus
Gavi: Gavi, the Vaccine Alliance
IHME: Institute of Health Metrics and Evaluation
ISO: International Organization of Standardization
SARA: Service Availability and Readiness Assessment
UNICEF: United Nations Children’s Fund
WHO: World Health Organization
WUENIC: WHO/UNICEF Estimates of National Immunization Coverage
